# FOXA2 Transcriptionally Activates SIRT1 to Inhibit Cochlear Ferroptosis in Noise-Induced Hearing Loss

**DOI:** 10.3390/genes17080953

**Published:** 2026-08-14

**Authors:** Xiaoru Dai, Peng Sun, Minyun Jiang, Lizhuang Xie, Hengdong Zhang, Baoli Zhu, Boshen Wang

**Affiliations:** 1 School of Public Health, Nanjing Medical University, Nanjing 211166, China; dxr614358@stu.njmu.edu.cn; 2 Institute of Occupational Disease Prevention, Jiangsu Provincial Center for Disease Control and Prevention, No. 172 Jiangsu Road, Nanjing 210009, China; xielizhuang@jscdc.cn (L.X.); hd-zhang@263.net (H.Z.); 3 Liyang Centre for Disease Control and Prevention, Changzhou 213300, China; sunpeng18252714192@163.com; 4 Jiading District Center for Patriotic Health and Health Promotion Guidance, Shanghai 201800, China; minyun3059@163.com

**Keywords:** SIRT1, FOXA2, SNP, ferroptosis, susceptibility, NIHL

## Abstract

**Background:** Noise-induced hearing loss (NIHL) is a pervasive occupational health challenge, yet the individual susceptibility mechanisms remain unclear. Ferroptosis, an iron-dependent form of programmed cell death, has been implicated in cochlear hair cell damage. This study aims to elucidate the regulatory role of the transcription factor FOXA2 and the deacetylase SIRT1 in ferroptosis and to investigate the association between *SIRT1* genetic polymorphisms and NIHL susceptibility in occupational populations. **Methods:** We employed a multi-level study design combining epidemiological investigation, animal models, and cellular experiments. First, a case–control study was conducted involving 1314 noise-exposed workers (639 cases vs. 675 controls) from a chemical fiber enterprise in Jiangsu Province to analyze the association between *SIRT1* single nucleotide polymorphisms (SNPs) and NIHL risk. Second, a C57BL/6J mouse model exposed to 120 dB white noise was established to assess cochlear morphology and protein expression. Third, in HEI-OC1 cochlear hair cells, we performed siRNA-mediated knockdown of *Foxa2* and dual-luciferase reporter assays to verify the transcriptional regulation of *SIRT1* and its downstream effects on the ferroptosis pathway. **Results:** Population analysis revealed that the *SIRT1* rs12778366 C allele was significantly associated with increased NIHL risk (OR = 1.386, 95% CI: 1.084–1.772, *p* = 0.009), and this association remained significant after adjustment (*p* = 0.041). Stratified analysis further revealed a significant gene–environment interaction in workers with >15 years of noise exposure (OR = 2.126, 95% CI: 1.401–3.226, *p* < 0.001). In vivo, noise exposure led to significant downregulation of *Sirt1* and *Foxa2* in cochlear tissues, accompanied by elevated ferroptosis markers (Fe^2+^, MDA) and depleted antioxidant defenses (GSH, xCT, and GPX4). Mechanistically, we demonstrate that FOXA2 transcriptionally activates *SIRT1* by binding to its promoter. Knockdown of FOXA2 in vitro suppressed *SIRT1* expression, suppressed xCT, a key component of the System Xc^−^/GPX4 antioxidant axis, and promoted ferroptosis-related cellular changes. **Conclusions:** This study identifies a novel protective axis where *FOXA2* prevents noise-induced ferroptosis in cochlear hair cells by transcriptionally upregulating SIRT1 and maintaining xCT-mediated antioxidant defense. Furthermore, the *SIRT1* rs12778366 polymorphism is identified as a candidate variant warranting further investigation in independent cohorts before clinical translation.

## 1. Introduction

Noise-induced hearing loss (NIHL) represents a prevalent and irreversible sensorineural impairment caused by prolonged exposure to high-intensity noise [1]. As the second most common form of sensorineural hearing loss globally, NIHL not only severely impacts the quality of life and mental health of workers but also imposes a substantial economic burden on healthcare systems and society [2,3]. Despite the widespread implementation of occupational protective measures, the incidence of NIHL remains high [4]. Epidemiological evidence suggests that NIHL is a complex disease resulting from the interplay between environmental exposures and individual factors. While noise intensity is the primary determinant, individual susceptibility varies significantly. Hearing outcomes vary substantially among workers with comparable noise exposure, indicating that environmental, clinical, and genetic factors may jointly influence NIHL susceptibility [5]. Confounding factors such as smoking, hypertension, and hyperlipidemia contribute to this variability, yet the underlying genetic predisposition remains a critical, albeit under-explored, determinant [6,7,8,9]. Therefore, identifying specific genetic biomarkers for early screening is urgent for the precision prevention of occupational NIHL.

The pathogenesis of NIHL is intricate, primarily involving mechanical damage and metabolic disorders within the cochlea. Excessive noise exposure triggers the overproduction of reactive oxygen species (ROS), leading to oxidative stress, inflammation, and ultimately, the death of cochlear hair cells (HCs) [10]. Since mammalian HCs are non-regenerative, their loss leads to permanent hearing deficits [11]. Recent studies have highlighted ferroptosis—an iron-dependent form of regulated cell death characterized by lipid peroxidation and mitochondrial shrinkage—as a key mechanism in ototoxicity and acoustic trauma [12,13]. Unlike apoptosis or necrosis, ferroptosis is driven by the failure of the glutathione peroxidase 4 (GPX4) antioxidant system and the accumulation of lipid reactive oxygen species. However, the specific molecular switches that regulate cochlear ferroptosis under noise stress are not fully elucidated.

SIRT1 (Sirtuin 1), a highly conserved NAD+-dependent deacetylase, is a key regulator of cellular metabolism, oxidative stress response, and aging [14]. Previous studies have demonstrated that SIRT1 exerts potent anti-inflammatory and antioxidant effects by deacetylating downstream targets, thereby protecting cells from stress-induced damage [15]. In the context of the auditory system, SIRT1 activity is essential for maintaining hair cell viability. Crucially, emerging evidence suggests a link between SIRT1 and the regulation of ferroptosis via the System Xc^−^/GPX4 axis, although this has rarely been verified in NIHL models [16]. Furthermore, the transcriptional regulation of *SIRT1* itself is critical. FOXA2 (Forkhead Box A2), a pioneer transcription factor, has been reported to regulate metabolic homeostasis and oxidative stress [17]. Bioinformatics analysis in our preliminary study indicated a potential regulatory site for FOXA2 in the *SIRT1* promoter region encompassing the rs12778366 polymorphism. This raises the hypothesis that FOXA2 may modulate NIHL susceptibility by regulating *SIRT1* expression and subsequent ferroptosis.

To verify this hypothesis, we employed an integrative study design that combines epidemiological analysis with in vivo and in vitro experimental validation. First, we conducted a case–control study in a Chinese occupational population to investigate the association between *SIRT1* single nucleotide polymorphisms (SNPs) and NIHL susceptibility. Second, we established noise-exposed mouse models and HEI-OC1 cell lines to elucidate the molecular mechanism by which the FOXA2/SIRT1 axis regulates ferroptosis. This study aims to provide novel mechanistic insights into noise-induced hair cell damage and to identify valid genetic biomarkers for the risk assessment of noise-exposed workers.

## 2. Materials and Methods

### 2.1. Study Population and Design

A cross-sectional case–control study was conducted involving employees from a large chemical fiber enterprise in Jiangsu Province, China. All participants consistently utilized standardized personal protective equipment (PPE), including earplugs and earmuffs, during their shifts. Inclusion criteria: (1) Han Chinese ethnicity; (2) employment duration ≥ 3 years; (3) complete occupational health surveillance records; (4) no history of otological diseases. Exclusion criteria: (1) diagnosis of organic ear diseases or conductive hearing loss; (2) family history of hereditary deafness; (3) history of head trauma or explosive deafness; (4) usage of ototoxic medications (e.g., aminoglycosides, cisplatin). Ultimately, a total of 1314 workers were recruited. Investigators underwent uniform training prior to data collection to ensure consistency. Written informed consent was obtained from all participants. This study was approved by the Ethics Committee of Jiangsu Provincial Center for Disease Control and Prevention (approval No. JSJK2025-B033-01) and conducted in accordance with the Declaration of Helsinki.

### 2.2. Environmental Noise Monitoring and Audiometric Assessment

Workplace noise intensity was measured in accordance with the Chinese National Standard GBZ/T 189.8-2007 (Measurement of Physical Factors in the Workplace–Part 8: Noise) [18]. For stationary posts, a Sound Level Meter (Quest SoundPro, TSI Quest, Oconomowoc, WI, USA) was employed; for mobile posts, a personal noise dosimeter (Quest NoisePro-DL, TSI Quest, Oconomowoc, WI, USA) was used. Measurements were taken at three randomly selected positions during varying operational times to calculate the time-weighted average (TWA) of noise exposure. The TWA noise exposure levels are reported in Table S5.

Pure-tone audiometry was performed using a calibrated audiometer in a sound-attenuating booth (background noise < 25 dB(A)) following the GB/T 16296.1-2018 standard [19]. Participants were required to be away from the noise environment for at least 16 h prior to testing. Pure-tone air-conduction thresholds were tested at 500, 1000, 2000, 3000, 4000, and 6000 Hz. Grouping Criteria: The audiometric data were adjusted for age and gender. Participants were classified into the case group (NIHL) if they exhibited a weighted value of the better ear > 26 dB [20]. The remaining participants were classified as the control group (normal hearing).

### 2.3. SNP Selection and Genotyping

The genomic location of the *SIRT1* gene was identified using the UCSC Genome Browser. Data from the “1000 Genomes Project” were analyzed to screen for potential single nucleotide polymorphisms (SNPs). Seven functional SNPs (rs10997868, rs12049646, rs12778366, rs2236319, rs2273773, rs3758391, and rs7895833) were selected based on the following criteria: (1) minor allele frequency (MAF) > 0.05; (2) adherence to Hardy–Weinberg equilibrium (HWE) (*p* > 0.05); (3) strong linkage disequilibrium (r^2^ > 0.8) within the haploblock; (4) documented disease relevance in literature over the past 15 years.

Genotyping: Genomic DNA was extracted from peripheral blood lymphocytes using the GoldMag^®^ Nanoparticle Universal DNA Purification Kit (GoldMag Co. Ltd., Xi’an, China) according to the manufacturer’s protocol. Genotyping was performed by Shanghai Biowing Applied Biotechnology Co., Ltd. (Shanghai, China) using the Ligase Detection Reaction–Polymerase Chain Reaction (LDR-PCR) method. The target DNA sequences were amplified using multiplex PCR, followed by the LDR reaction. The ligation products were separated using an ABI 3730XL DNA Analyzer (Applied Biosystems, Foster City, CA, USA). Data analysis and haplotype construction were performed using GeneMapper 4.1 (Applied Biosystems, Foster City, CA, USA) and Haploview 4.2 (Broad Institute, Cambridge, MA, USA), respectively.

### 2.4. Animal Model and Noise Exposure

Male C57BL/6J mice (6–8 weeks old; 18–22 g) were purchased from GemPharmatech Co., Ltd. (Nanjing, China). All animal procedures were approved by the Animal Ethics Committee of Jiangsu Provincial Center for Disease Control and Prevention (JSJK/JL-161) and complied with relevant ethical animal welfare regulations. Mice were housed under controlled conditions (12 h light/dark cycle, 22 ± 2 °C) with background noise < 60 dB SPL. Mice were randomly divided into a control group (*n* = 5) and a noise-exposure group (*n* = 5). The five mice in each group constituted the independent biological replicates for the in vivo experiments. For quantitative assays performed in technical triplicate, the three technical measurements from each mouse were averaged to obtain one animal-level value for statistical analysis. The noise group was exposed to broadband white noise at 120 dB SPL for 2 h daily for 2 consecutive days. Noise was generated using Cool Edit Pro V2.1 (Syntrillium Software Corporation, Phoenix, AZ, USA) and delivered via loudspeakers (gkb-60a, Yamei, Shanghai, China) positioned 10 cm from the cage within a soundproof chamber. Sound levels were calibrated using a sound level meter (AWA5630, Aihua Instruments, Hangzhou, China) to ensure uniformity (variation < 1 dB).

### 2.5. Auditory Brainstem Response (ABR) Measurement

Cochlear function was assessed via ABR post-exposure. Mice were anesthetized with 1% sodium pentobarbital (Sigma-Aldrich, St. Louis, MO, USA; 100 mg/kg, i.p.) and placed on a heating pad to maintain body temperature. Subdermal needle electrodes were placed at the vertex (active), mastoid (reference), and hind leg (ground). ABR thresholds were recorded in response to tone bursts (5 ms duration, 0.5 ms rise/fall time) at frequencies of 4, 8, 16, 24, and 32 kHz using a TDT System III workstation (Tucker-Davis Technologies, Alachua, FL, USA). The response threshold was determined as the lowest stimulus level that evoked a visually replicable waveform (wave III).

### 2.6. Tissue Collection and Biochemical Analysis

Blood Sampling: Blood was collected via the retro-orbital route. Serum was separated by centrifugation, and leukocytes were isolated. Levels of Fe^2+^, glutathione (GSH), oxidized glutathione (GSSG), and malondialdehyde (MDA) were measured using commercial ELISA kits (Nanjing Jiancheng Bioengineering Institute, Nanjing, China) following the manufacturer’s protocols.

Cochlear Harvesting: Mice were sacrificed by cervical dislocation. Cochleae were rapidly dissected under a stereomicroscope (Dumont SA, Montignez, Switzerland). Tissue was processed for either RNA/protein extraction or immunofluorescence analysis. Protein levels of FOXA2, SIRT1, xCT, G6PD, and GPX4 in cochlear samples were quantified using ELISA kits according to the manufacturers’ instructions.

Cochlear whole-mount immunofluorescence staining: Cochleae were rinsed with PBS, and the cochlear apex was perforated to expose the round and oval windows. The cochleae were perfused with 4% paraformaldehyde, prepared from a 16% paraformaldehyde stock solution (Shanghai Chunlian Biotechnology Co., Ltd., Shanghai, China), and fixed in 4% paraformaldehyde for 1 h at room temperature, followed by decalcification in 0.12 M EDTA for 2 days. The basilar membrane was carefully dissected and mounted on Cell-Tak-coated 10 mm coverslips (Corning Inc., Corning, NY, USA). Samples were blocked for 1 h at room temperature using MAXblock™ Blocking Medium (Active Motif, Carlsbad, CA, USA) and incubated with the primary antibody overnight at 4 °C, followed by incubation with the fluorescent secondary antibody for 1 h at room temperature. Nuclei were counterstained with DAPI (Sigma-Aldrich, St. Louis, MO, USA), and the samples were mounted and imaged. Hair cells were labeled with Myosin VIIa, and F-actin was labeled with phalloidin.

### 2.7. Cell Culture and Transfection

The HEI-OC1 cochlear hair cell line (kindly donated by F. Kalinec at the House Ear Institute in Los Angeles, CA, USA) was cultured in high-glucose DMEM (Gibco, Grand Island, NY, USA) supplemented with 10% fetal bovine serum (FBS; Clark Bioscience, Webster, TX, USA) at 37 °C in a 5% CO_2_ humidified atmosphere.

Plasmids and siRNA: *Foxa2* Knockdown: Three *Foxa2*-specific small interfering RNAs (siRNAs) were synthesized by Guangzhou RiboBio Co., Ltd. (Guangzhou, China).

Luciferase Vectors: Primer sequences for the *Foxa2* promoter and *SIRT1* rs12778366 region were synthesized by Shanghai Kehua Bio-engineering Co., Ltd. (Shanghai, China). The target fragments were cloned into the pGL3-Basic or pcDNA3.1 vector (Promega, Madison, WI, USA). The FOXA2 overexpression plasmid and reporter gene vectors are shown in Figures S2 and S3, respectively, and their sequencing results are provided in Tables S2 and S3.

Transfection: Cells were seeded in 6-well plates and transfected using Lipofectamine 2000 (Invitrogen, Carlsbad, CA, USA) according to the manufacturer’s instructions. The detailed transfection system is provided in Table S4. Cells were harvested 48 h post-transfection for subsequent analysis.

### 2.8. Dual-Luciferase Reporter Assay

To validate the transcriptional regulation of *SIRT1* by FOXA2, a dual-luciferase reporter assay was performed. HEI-OC1 cells were co-transfected with the constructed reporter plasmids containing wild-type or mutant *SIRT1* promoter regions and FOXA2 overexpression vectors. Luciferase activity was measured 48 h post-transfection using the Dual-Luciferase^®^ Reporter Assay System (Promega, Madison, WI, USA) on a microplate reader (SpectraMax i3, Molecular Devices, San Jose, CA, USA). Relative luciferase activity was normalized to Renilla luciferase activity.

### 2.9. qRT-PCR and Western Blot

qRT-PCR: Total RNA was extracted using TRIzol reagent (Life Technologies, Carlsbad, CA, USA). cDNA was synthesized using the PrimeScript™ RT Reagent Kit (Takara, Dalian, China). Real-time PCR was performed using TB Green^®^ Premix Ex Taq™ II (Takara, Dalian, China) on an ABI 7500 Real-Time PCR System (Applied Biosystems, Foster City, CA, USA). The thermal cycling conditions were 95 °C for 30 s, followed by 40 cycles of 95 °C for 5 s and 60 °C for 34 s. Primer sequences are listed in Table S1.

Western Blot: Protein concentrations were determined using a BCA Protein Assay Kit (Beyotime, Shanghai, China). Lysates (30 µg) were separated by 10% SDS-PAGE and transferred to PVDF membranes (Millipore, Billerica, MA, USA). Membranes were blocked with 5% non-fat milk and then physically sectioned according to the expected molecular-weight regions of the target proteins before antibody incubation and chemiluminescent imaging. Individual membrane strips were subsequently incubated with the corresponding primary and secondary antibodies and imaged separately. Therefore, complete full-membrane chemiluminescent images spanning the entire molecular-weight range were not generated. The membrane strips were incubated overnight at 4 °C with primary antibodies: Anti-SIRT1 (1:1000, Cell Signaling Technology, #9475, Danvers, MA, USA), Anti-FOXA2 (1:1000, Abcam, ab108422, Cambridge, UK), Anti-xCT (1:1000, Abcam, ab37185), Anti-GPX4 (1:1000, Abcam, ab125066), Anti-G6PD (1:1000, Proteintech, 25413-1-AP, Wuhan, China), and Anti-β-actin (1:5000, Proteintech, 66009-1-Ig). The secondary antibodies used were HRP-conjugated goat anti-rabbit/mouse IgG (1:5000, Proteintech). Bands were visualized using an ECL detection system (Thermo Fisher Scientific, Waltham, MA, USA). Band intensities were quantified by densitometric analysis, and target-protein signals were normalized to β-actin.

### 2.10. Assessment of Ferroptosis In Vitro

To induce ferroptosis, HEI-OC1 cells were treated with Erastin (10 µM for 24 h; Selleckchem, Houston, TX, USA). Ferrostatin-1 (Fer-1; 1 µM; Meilunbio, Dalian, China) was added 2 h before erastin and maintained during the subsequent 24 h erastin exposure. The primary mechanistic comparison was performed between the si-NC and si-FOXA2 groups, and cells were harvested 48 h after transfection. Intracellular Fe^2+^ levels and lipid peroxidation markers (MDA, GSH, GSSG) were quantified using ELISA kits as previously described. In the si-NC and si-FOXA2 comparison, xCT, G6PD, and GPX4 protein levels were assessed by Western blotting and quantified by densitometric analysis as described above. Cell ultrastructure was examined using a transmission electron microscope (Hitachi H-7650, Tokyo, Japan). Samples were processed by Nanjing Yuan Cell Technology Co., Ltd. (Nanjing, China).

### 2.11. Statistical Analysis

Statistical analyses were performed using IBM SPSS Statistics v25.0 (IBM Corp., Armonk, NY, USA). Quantitative data are expressed as mean ± standard deviation (SD). Differences between two groups were analyzed using Student’s *t*-test. For multiple group comparisons, one-way ANOVA was used, followed by an LSD post hoc test (for equal variances) or Dunnett’s T3 test (for unequal variances). A *p*-value < 0.05 was considered statistically significant. To account for correlations among the seven tested *SIRT1* SNPs, the effective number of independent tests was estimated using the eigenvalue-based method of Gao et al. [21]. Multiple-testing-adjusted *p*-values were calculated based on the resulting effective number of tests; both unadjusted and adjusted *p*-values are reported in Table S7. A post hoc power calculation for the rs12778366 association was performed using Quanto 1.2.4 (University of Southern California, Los Angeles, CA, USA; case–control design, MAF = 0.095, two-sided α = 0.05, observed OR = 1.386), yielding an estimated power of 74.2%.

## 3. Results

### 3.1. Demographic Characteristics and Genotypic Screening

A total of 1314 workers were recruited for this study, comprising 639 individuals in the case group (NIHL) and 675 in the control group (normal hearing). As summarized in Supplementary Table S5, there were no statistically significant differences between the two groups regarding age, gender, smoking habits, alcohol consumption, duration of noise exposure, or noise intensity (*p* > 0.05), indicating that the baseline characteristics were well-balanced and comparable. However, the average high-frequency hearing threshold in the case group (37.78 ± 12.11 dB) was significantly higher than that of the control group (19.50 ± 8.65 dB; *p* < 0.001), confirming the validity of the grouping. We subsequently genotyped seven candidate SNPs of the *SIRT1* gene, and the genotype distributions for all loci conformed to the Hardy–Weinberg equilibrium (*p* > 0.05, Table S6). Among the tested loci, the genotype distribution of rs12778366 differed significantly between the case and control groups, which remained significant after correction for multiple testing using the effective-number-of-tests approach (adjusted *p* = 0.041, Table S7). The other six loci showed no significant differences. Consequently, rs12778366 was selected for further analysis.

### 3.2. Association Analysis Between SIRT1 Polymorphisms and NIHL Susceptibility

Logistic regression analysis showed nominal associations between the SIRT1 rs12778366 polymorphism and NIHL risk (Table S8). In the codominant model, the CT genotype was nominally associated with increased risk compared to the TT genotype (adjusted OR = 1.328, 95% CI: 1.014–1.740, *p* = 0.039). The CC genotype showed a larger point estimate but with wide confidence intervals due to low genotype frequency (adjusted OR = 4.548, 95% CI: 0.959–21.568, *p* = 0.056). In the dominant model, CT + CC carriers had nominally higher risk than TT carriers (adjusted OR = 1.380, 95% CI: 1.057–1.801, *p* = 0.018). In the recessive model, CC homozygotes showed elevated risk relative to TT + CT carriers, though the estimate was imprecise (adjusted OR = 4.298, 95% CI: 0.907–20.364, *p* = 0.066). The allele model showed that the C allele was nominally associated with increased NIHL risk (adjusted OR = 1.386, 95% CI: 1.084–1.772, *p* = 0.009). To further explore the gene–environment interaction, we performed a stratified analysis (Tables S9 and S10). In workers with > 15 years of noise exposure, carriers of the risk-associated C allele (CC + CT) had a significantly higher risk of NIHL compared to TT carriers. In stratified analysis (Tables S9 and S10), the association between the C allele and NIHL was more pronounced in workers with >15 years of noise exposure (CT + CC vs. TT: OR = 2.126, 95% CI: 1.401–3.226, *p* < 0.001) and in those exposed to >85 dB(A) noise intensity (CT + CC vs. TT: OR = 1.664, 95% CI: 1.115–2.482, *p* = 0.012), suggesting a potential gene–environment interaction. Additional SNP–environment interaction analyses are presented in Table S11.

### 3.3. Verification of Cochlear Damage and Ferroptosis in Noise-Exposed Mice

**Figure 1:**
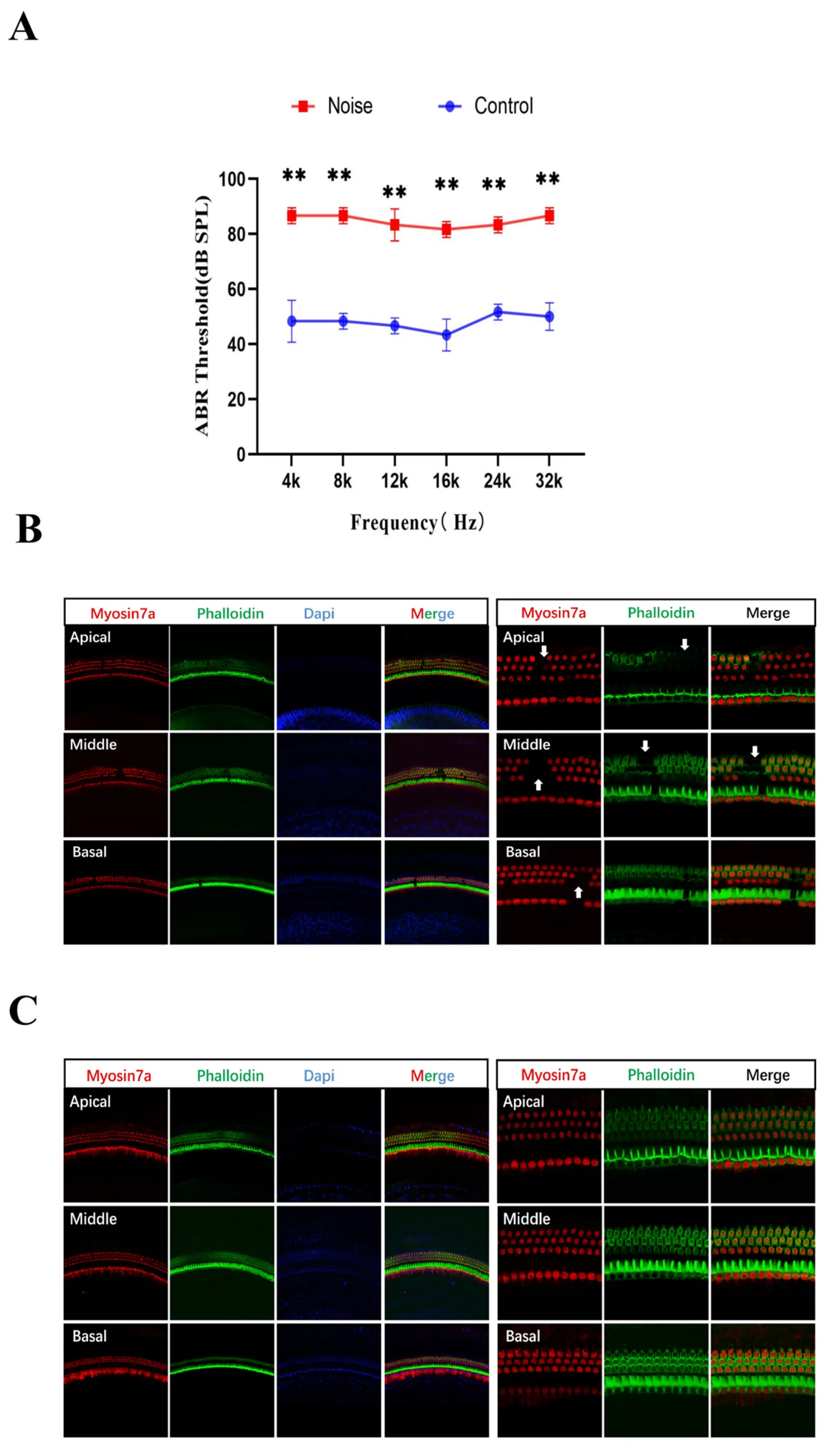
Results of auditory brainstem response (ABR) test and fluorescence immunoassay: (**A**) ABR thresholds at 4, 8, 16, 24, and 32 kHz in control (*n* = 5) and noise-exposed (*n* = 5) mice. Data are mean ± SD. ** *p* < 0.01. (**B**) The representative cochlear whole-mount images from the noise-exposed group. On the right is a 63-fold magnification; “→” indicates the missing and damaged cochlear hair cells. (**C**) The representative images from the control group. Scale bar: 20 μm.

**Figure 2:**
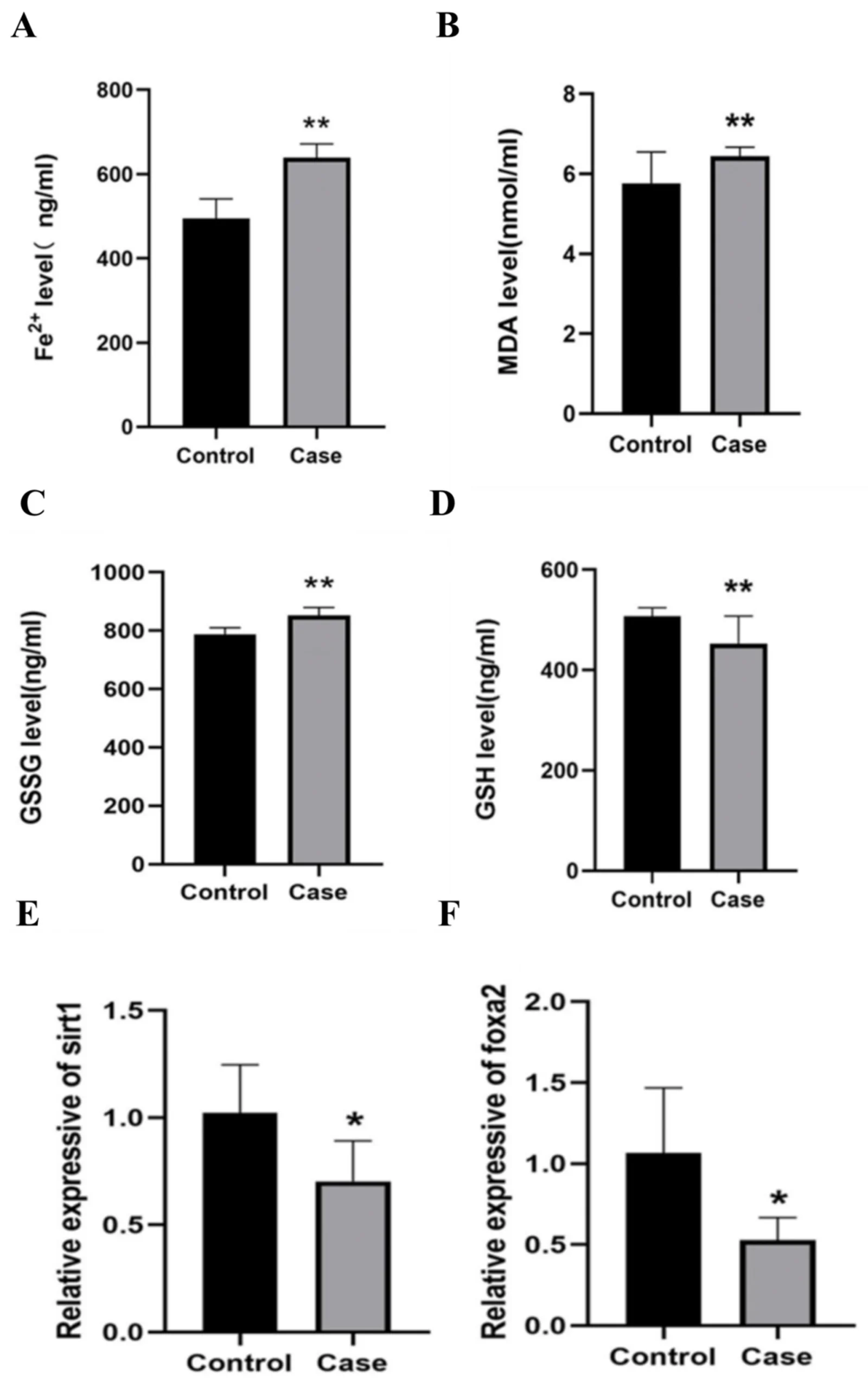
Serum ferroptosis markers and cochlear mRNA expression in control and noise-exposed mice: (**A**) Serum Fe^2+^ concentrations. (**B**) Serum malondialdehyde (MDA) levels. (**C**) Serum oxidized glutathione (GSSG) levels. (**D**) Serum reduced glutathione (GSH) levels. (**E**) Relative *Sirt1* mRNA expression in cochlear tissue. (**F**) Relative *Foxa2* mRNA expression in cochlear tissue. Data are mean ± SD (*n* = 5 per group). * *p* < 0.05, ** *p* < 0.01 versus control.

**Figure 3:**
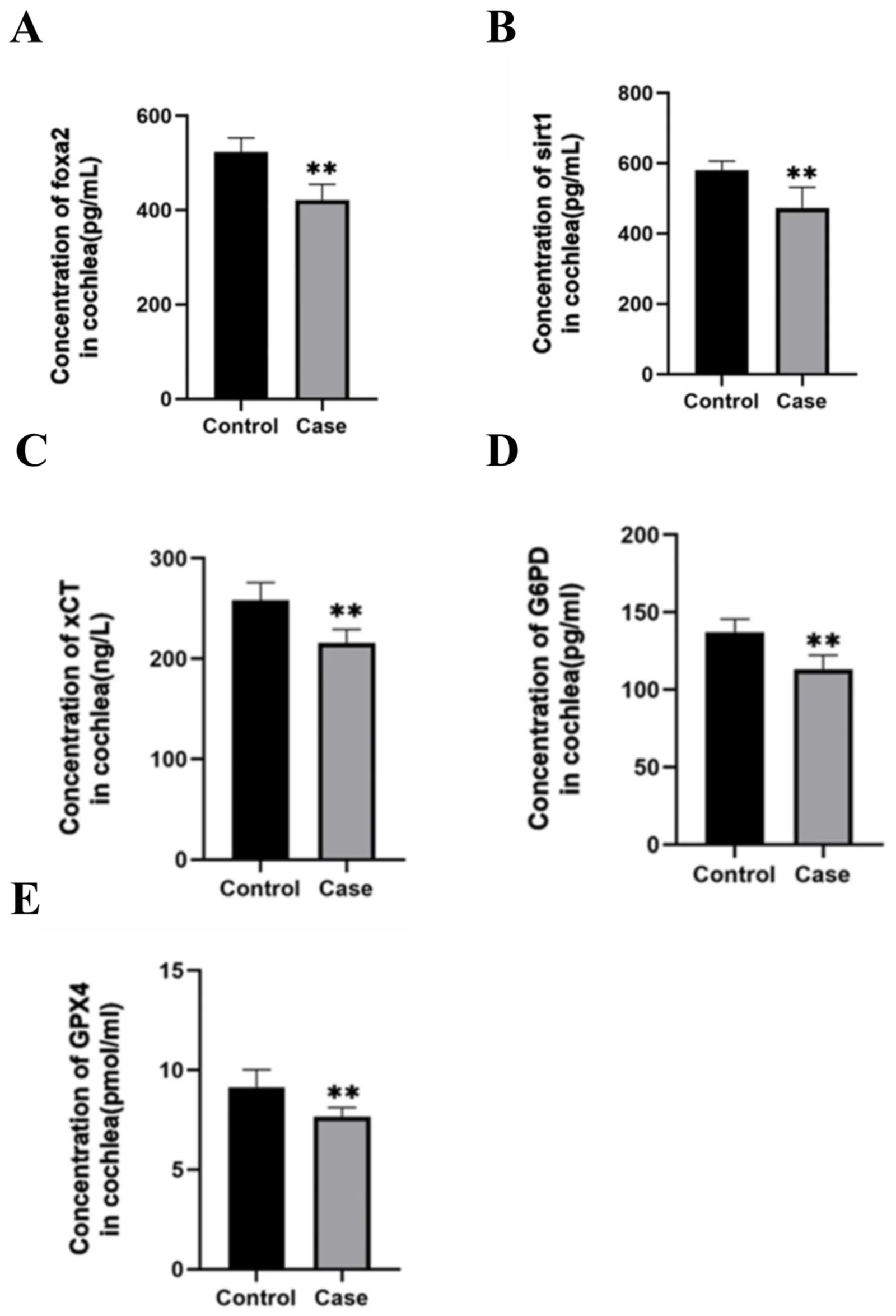
Ferroptosis-related protein expression in mouse cochlear tissue after noise exposure: (**A**) FOXA2 protein levels. (**B**) SIRT1 protein levels. (**C**) xCT (SLC7A11) protein levels. (**D**) G6PD protein levels. (**E**) GPX4 protein levels. Protein levels were quantified by ELISA. Data are mean ± SD (*n* = 5 per group). ** *p* < 0.01 versus control.

To validate the epidemiological findings in vivo, we established a mouse model of noise-induced hearing loss. Post-exposure ABR testing showed significantly elevated hearing thresholds across all tested frequencies (4–32 kHz) in the noise group compared to controls (*p* < 0.05, Figure 1A). Consistent with this functional impairment, representative cochlear whole-mount images showed disorganization of the outer hair cell rows and apparent focal discontinuities in the noise-exposed group, particularly in the middle cochlear turn (Figure 1B,C). Serological analysis (Figure 2) further demonstrated that noise exposure led to a systemic oxidative imbalance, characterized by significantly increased levels of Fe^2+^, MDA (a lipid peroxidation marker), and GSSG, along with a marked reduction in the antioxidant GSH (*p* < 0.05). At the tissue level, *Sirt1* and *Foxa2* mRNA levels and SIRT1 and FOXA2 protein levels were significantly downregulated in the cochleae of noise-exposed mice compared to controls (*p* < 0.05, Figure 2 and Figure 3). Furthermore, key ferroptosis-related proteins, including xCT (*p* < 0.01), G6PD (*p* < 0.05), and GPX4 (*p* < 0.05), were significantly decreased in the noise-exposed group, supporting the hypothesis that noise exposure triggers cochlear ferroptosis via the suppression of these antioxidant pathways.

### 3.4. Transcriptional Regulation of SIRT1 by FOXA2

**Figure 4:**
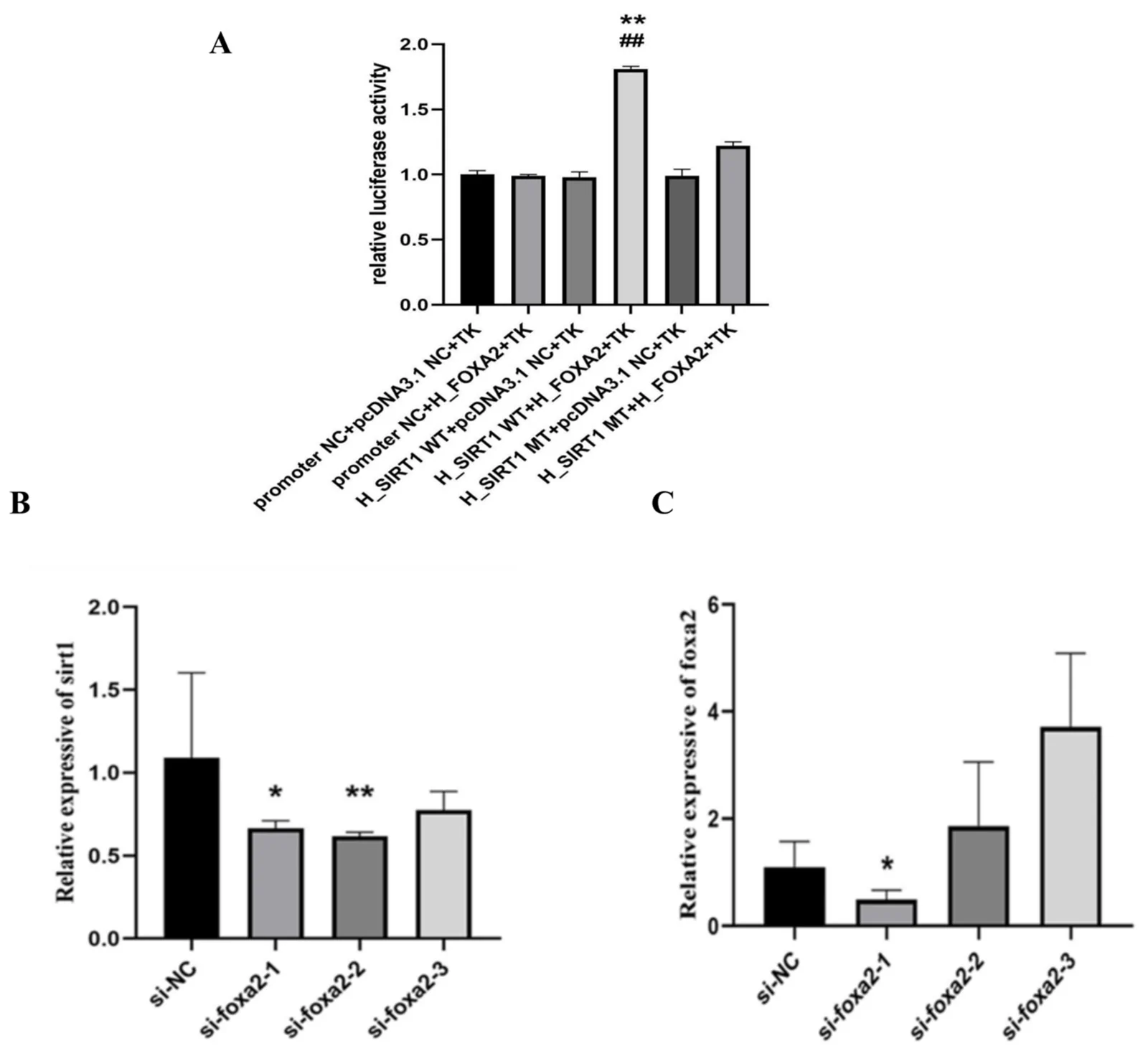
Dual-luciferase reporter assay of FOXA2-dependent *SIRT1* promoter regulation and siRNA knockdown efficiency screening: (**A**) Relative luciferase activity of HEI-OC1 cells co-transfected with WT-T or MT-C *SIRT1* promoter constructs (containing the rs12778366 T or C allele, respectively) together with FOXA2 overexpression (FOXA2 + TK) or control (pcDNA3.1NC) vectors. Data are mean ± SD from three independent experiments. ** in H_SIRT1 WT + H_FOXA2 + TK and promoter, compared between NC + H_FOXA2 + TK and H_SIRT1 MT + H_FOXA2 + TK, *p* < 0.05; ## indicates the comparison between H_SIRT1 WT + H_FOXA2 + TK and H_SIRT1 WT + pcDNA3.1NC, *p* < 0.05. (**B**) Relative *Sirt1* mRNA expression after transfection with three *Foxa2*-specific siRNAs. (**C**) Relative *Foxa2* mRNA expression after siRNA transfection. * *p* < 0.05, ** *p* < 0.01 versus si-NC.

Bioinformatics analysis identified multiple potential transcription factor binding sites in the *SIRT1* promoter region, with the transcription factor FOXA2 yielding the highest binding score specifically at the region containing the rs12778366 polymorphism (Figure S1). To verify this regulatory relationship, we performed a dual-luciferase reporter assay (Figure 4). The results showed that overexpression of FOXA2 significantly enhanced the luciferase activity of the reporter vector containing the wild-type T allele of rs12778366 (*p* < 0.05). In contrast, *FOXA2* overexpression failed to significantly induce the activity of the vector containing the mutant C allele. These findings suggest that the rs12778366 T allele facilitates FOXA2-dependent transcriptional activation of the *SIRT1* promoter, whereas the risk-associated C allele reduces this FOXA2-dependent transcriptional activity.

### 3.5. FOXA2/SIRT1 Axis Regulates Ferroptosis in HEI-OC1 Cells

**Figure 5:**
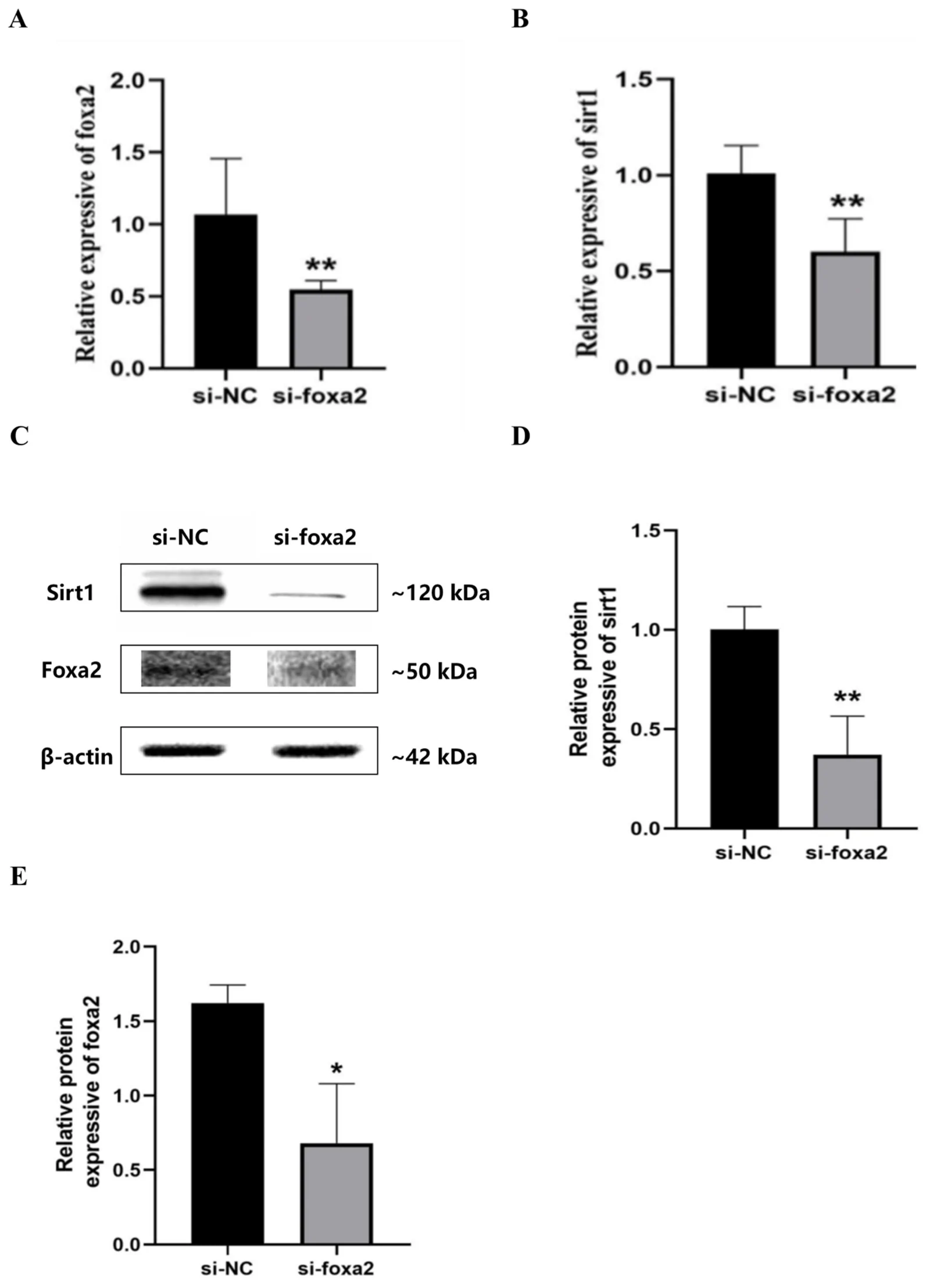
Effect of *Foxa2* knockdown on *Sirt1* and *Foxa2* mRNA and protein expression in HEI-OC1 cells: (**A**) *Foxa2* mRNA expression after siRNA-mediated knockdown. (**B**) *Sirt1* mRNA expression after *Foxa2* knockdown. (**C**) Representative Western blot images of FOXA2, SIRT1, and β-actin (loading control). (**D**) Densitometric quantification of SIRT1 protein expression normalized to β-actin. (**E**) Densitometric quantification of FOXA2 protein expression normalized to β-actin. Data are presented as mean ± SD. * *p* < 0.05, ** *p* < 0.01 versus si-NC.

**Figure 6:**
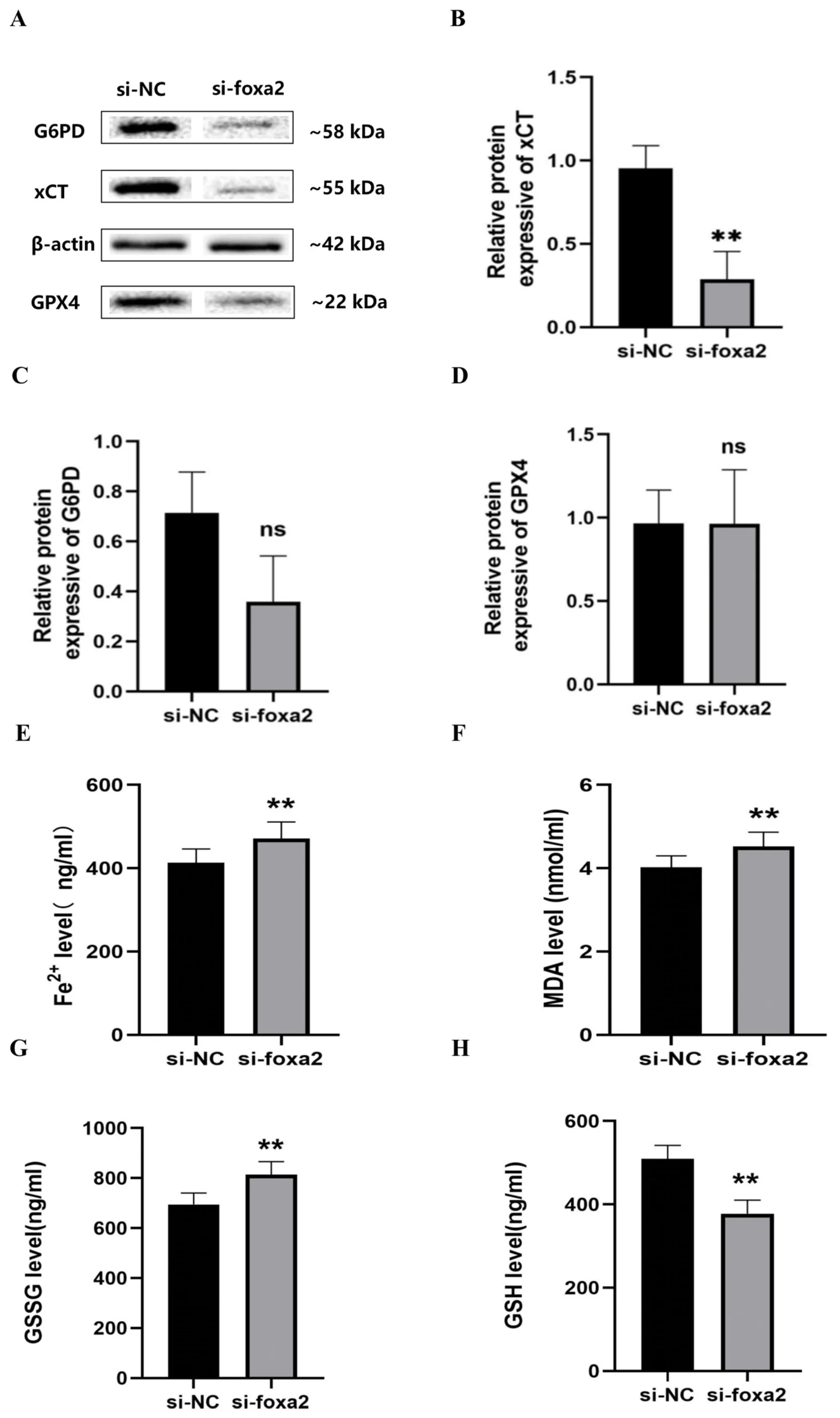
Ferroptosis-related protein expression and biochemical markers after *Foxa2* knockdown in HEI-OC1 cells: (**A**) Representative Western blot images of xCT, G6PD, GPX4, and β-actin. The target proteins were analyzed in separate Western blot runs. (**B**–**D**) Densitometric quantification of (**B**) xCT, (**C**) G6PD, and (**D**) GPX4 protein expression normalized to β-actin. (**E**–**H**) Levels of (**E**) intracellular Fe^2+^, (**F**) MDA, (**G**) GSSG, and (**H**) GSH after si-FOXA2 knockdown. Data are presented as mean ± SD. ** *p* < 0.01 versus si-NC; ns, not significant (*p* > 0.05).

To confirm the causal mechanism, we performed siRNA-mediated knockdown of *Foxa2* in HEI-OC1 cochlear hair cells. Among the three synthesized siRNAs, si-FOXA2-1 demonstrated the highest knockdown efficiency and was selected for subsequent experiments (Figure 4). Knockdown of *Foxa2* led to a significant reduction in both *SIRT1* mRNA and protein levels (*p* < 0.05, Figure 5), confirming that SIRT1 is a downstream target of FOXA2 in cochlear cells. Western blot densitometric analysis showed that *FOXA2* knockdown significantly reduced xCT protein levels (*p* < 0.01), whereas G6PD and GPX4 showed decreasing trends that did not reach statistical significance (*p* > 0.05; Figure 6). Phenotypically, following FOXA2 knockdown, cells exhibited hallmarks of ferroptosis, including significantly elevated intracellular Fe^2+^ and MDA levels and depleted GSH (*p* < 0.05).

## 4. Discussion

Clinical observations consistently reveal significant heterogeneity in hearing outcomes among workers exposed to identical noise environments, suggesting that the “equal energy hypothesis”—which posits that damage is strictly proportional to noise energy—is insufficient to fully explain the pathogenesis of auditory impairment [22,23,24]. This discrepancy underscores a complex interplay between environmental exposure and individual genetic predisposition. By employing an integrative multi-level framework, our study bridges this gap and offers converging evidence that NIHL heterogeneity is biologically driven. Specifically, we identify the *SIRT1* rs12778366 polymorphism as a candidate modulator of cochlear defense that may contribute to ferroptosis susceptibility—a specific form of regulated cell death.

Historically, hair cell loss was attributed primarily to apoptosis or necrosis [25]. However, emerging evidence from the 2023–2025 period has refined our understanding, identifying ferroptosis—an iron-dependent, lipid peroxidation-driven cell death—as a critical mechanism in sensorineural hearing loss [12,13,26,27]. Unlike apoptosis, which is an energy-dependent process, ferroptosis is characterized by the catastrophic failure of the antioxidant defense system, leading to membrane rupture [28]. In our study, noise-exposed mice exhibited the classic hallmarks of ferroptosis: shrunken mitochondria with increased membrane density, systemic iron overload (elevated Fe^2+^), and the accumulation of toxic lipid peroxides (MDA). Crucially, we observed a synchronous depletion of GPX4 and xCT (SLC7A11), key regulators of ferroptosis. These findings align with recent transcriptomic analyses showing that ferroptosis-related genes are significantly enriched in cisplatin- and noise-damaged cochleae [29]. Therefore, we propose that under high-intensity noise stress, the cochlear cellular environment shifts toward ferroptotic stress, highlighting xCT/GPX4 as potential therapeutic targets.

A key contribution of this work is the identification of the FOXA2/SIRT1 axis as an important regulator of cochlear ferroptosis. While SIRT1 is a well-established guardian of mitochondrial function and redox balance in the inner ear, its upstream regulation has remained elusive [30,31,32]. Here, we demonstrate that the pioneer transcription factor FOXA2 transcriptionally regulates *SIRT1* through its promoter region, as supported by our dual-luciferase reporter assay data. Our findings are supported by parallel mechanisms observed in oncology, where FOXA2 suppression has been shown to induce ferroptosis via the NRF2/GPX4 pathway in colorectal and gastric cancers [33,34,35]. We posit that a similar protective mechanism exists in the auditory system: FOXA2 maintains basal SIRT1 levels, which in turn stabilize the xCT/GPX4 axis to neutralize lipid ROS. Noise exposure disrupts this axis by downregulating FOXA2, thereby “releasing the brake” on ferroptosis. To our knowledge, this is among the first studies to implicate FOXA2 in auditory protection, expanding its known functions beyond metabolic regulation. We note that direct chromatin-level binding evidence would further strengthen the conclusion of FOXA2-*SIRT1* promoter interaction; the present luciferase data demonstrate FOXA2-dependent transcriptional regulation of the *SIRT1* reporter construct. Notably, the relative preservation of G6PD, which generates NADPH for glutathione regeneration via the pentose phosphate pathway, suggests that FOXA2 knockdown primarily impairs cystine import through xCT downregulation rather than disrupting NADPH-dependent GSH recycling. This is consistent with the observation that cysteine availability, rather than NADPH supply, is typically rate-limiting for GSH synthesis [36,37].

Connecting our molecular data to the epidemiological results, we propose a “Broken Switch” hypothesis to explain the genetic susceptibility to NIHL. Our case–control study identified the rs12778366 C allele as a significant risk factor (adjusted OR = 1.386, 95% CI: 1.084–1.772, *p* = 0.009). The biological plausibility of this association was functionally supported by our dual-luciferase assays: the risk-associated C allele disrupts the high-affinity binding motif for FOXA2, leading to intrinsically lower transcriptional activity of *SIRT1*. Consequently, carriers of the C allele likely possess a “hypomorphic” antioxidant defense system. Under normal acoustic conditions, this deficit may be compensated; however, under the metabolic demand of chronic noise exposure (>15 years, as seen in our stratified analysis), this fragile defense collapses, triggering premature ferroptosis [38]. We propose a “Broken Switch” model wherein the rs12778366 C allele disrupts a high-affinity FOXA2 binding motif, leading to intrinsically lower *SIRT1* transcriptional activity; carriers of the C allele may therefore possess a constitutively compromised antioxidant defense that collapses under sustained noise stress. Future studies in diverse populations are warranted to validate the generalizability of this model.

The identification of *SIRT1* rs12778366 as a functional biomarker has potential translational implications if independently validated [39,40,41]. Current occupational health frameworks rely heavily on “one-size-fits-all” protection (e.g., noise limits < 85 dB). Our findings suggest that the *SIRT1* rs12778366 variant may, after independent replication, contribute to multifactorial risk stratification models. If confirmed in prospective studies, C allele carriers might benefit from enhanced surveillance; however, the clinical utility of genetic screening for NIHL prevention remains to be established. Several limitations should be considered. The cross-sectional, single-enterprise cohort consisted predominantly of male Han Chinese workers, which limits causal inference and broader generalizability. The animal study used an acute high-intensity noise model and a relatively small sample size, and the in vivo findings should therefore be regarded as exploratory. Moreover, ferroptosis was evaluated mainly through biochemical and ultrastructural indicators, while the FOXA2-*SIRT1* regulatory relationship was supported by reporter assays rather than direct chromatin-binding experiments. Larger prospective cohorts and additional mechanistic validation are needed to confirm the genetic association and the proposed regulatory mechanism.

## 5. Conclusions

In summary, this study identifies a regulatory pathway wherein the *SIRT1* rs12778366 polymorphism modulates NIHL susceptibility through altered FOXA2-mediated transcription of *SIRT1*. Our data indicate that dysregulation of the FOXA2/SIRT1 axis is a contributing mechanism to noise-induced ferroptosis in cochlear hair cells. These findings provide insights into the molecular basis of individual susceptibility and suggest that the ferroptosis pathway may represent a candidate target for future preventive strategies.

## Data Availability

The data presented in this study are available upon request from the corresponding authors due to the protection of participant privacy and strict confidentiality agreements.

## Supplementary Materials

The following supporting information can be downloaded at https://www.mdpi.com/article/10.3390/genes17080953/s1. Figure S1: Schematic representation of the relationship between SNPs and SIRT1 gene location; Figure S2: FOXA2 overexpression plasmid; Figure S3: Reporter gene vectors; Table S1: qRT-PCR Primer Sequences; Table S2: Sequencing results of FOXA2 overexpression vector; Table S3: Sequencing results of reporter gene vectors; Table S4: Cell transfection system; Table S5: Basic Characteristics of the Study Population; Table S6: Basic information about SNPs sites and Hardy-Weinberg tests; Table S7: Genotype distributions of the seven candidate SNPs in NIHL cases and controls; Table S8: Association between rs12778366 and NIHL susceptibility under different genetic models; Table S9: Stratified analyses of the association between gene polymorphisms and susceptibility to NIHL (dominant model); Table S10: Interactions between gene polymorphisms and environmental factors; Table S11: SNPs interaction analysis.
