# Supplementary material for: FOXA2 Transcriptionally Activates SIRT1 to Inhibit Cochlear Ferroptosis in Noise-Induced Hearing Loss
Source: Genes (Basel). 2026 Aug 14;17(8):953. doi: 10.3390/genes17080953 (PMC13511884; doi:10.3390/genes17080953)
Supplement: Supplementary file 1 [file genes-17-00953-s001.zip › genes-4446311-supplementary.pdf]

Supplementary Material

1 Supplementary Figures and Tables

1.1 Supplementary Figures

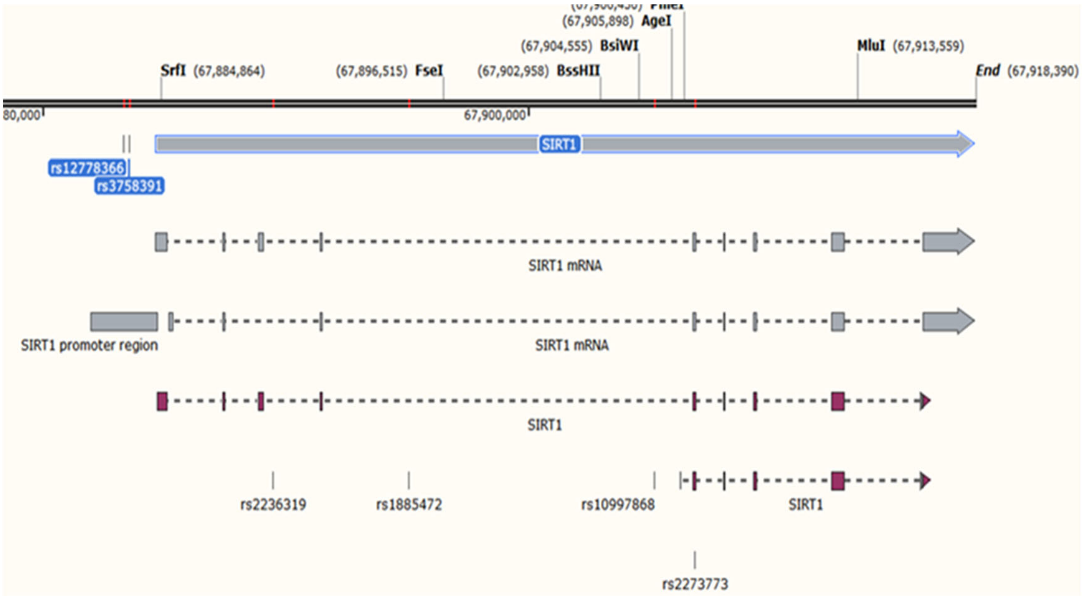

Supplementary Figure S1. Schematic representation of the relationship between SNPs and SIRT1 gene location

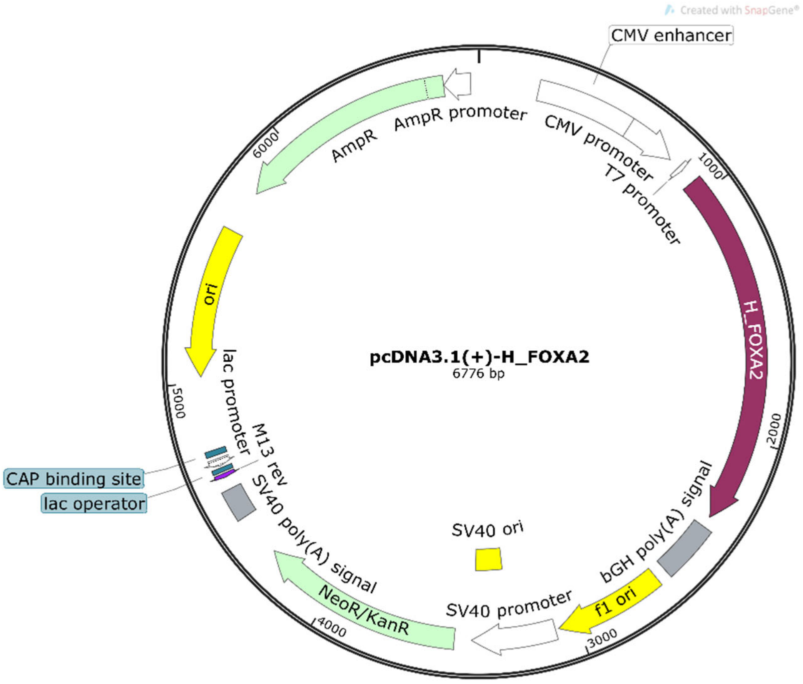

Supplementary Figure S2. FOXA2 overexpression plasmid

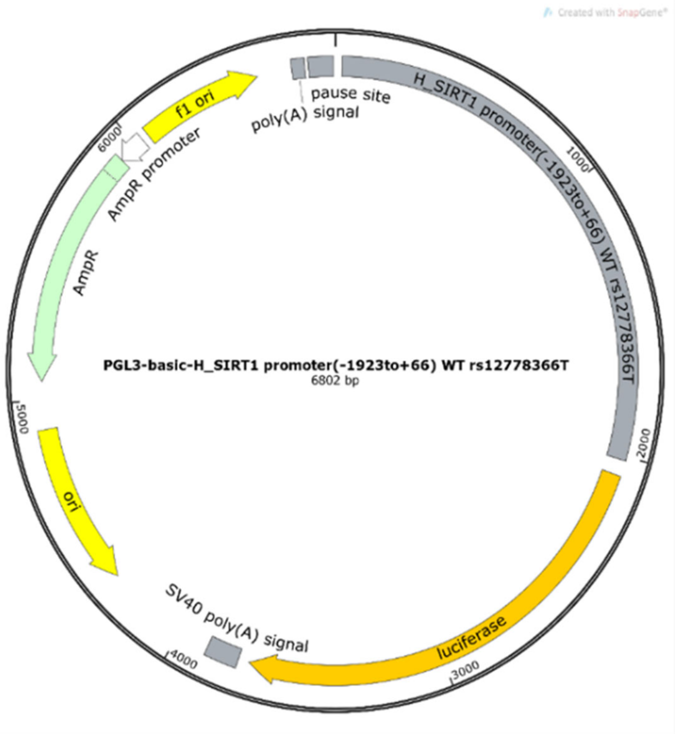

WT is rs12778366 wild type, MT is rs12778366 mutant.

Supplementary Figure S3. Reporter gene vectors

1.2 Supplementary Tables

Supplementary Table S1. qRT-PCR Primer Sequences

Gene	Direction	Sequence (5′→3′)
Sirt1	Forward	TGATTGGCACCGATCCTCG
	Reverse	CCACAGCGTCATATCATCCAG
Foxa2	Forward	TCCGACTGGAGCAGCTACTAC
	Reverse	GCGCCCACATAGGATGACA
β-actin	Forward	GGGAAATCGTGCGTGAC
	Reverse	AGGCTGGAAAAGAGCCT

Supplementary Table S2. Sequencing results of FOXA2 overexpression vector

Plasmids: pcDNA3.1(+)-H_FOXA2

GCCACCATGCACTCGGCTTCCAGTATGCTGGGAGCGGTGAAGATGGAAG
GGCACGAGCCGTCCGACTGGAGCAGCTACTATGCAGAGCCCGAGGGCT
ACTCCTCCGTGAGCAACATGAACGCCGGCCTGGGGATGAACGGCATGA
ACACGTACATGAGCATGTCGGCGGCCGCCATGGGCAGCGGCTCGGGCA
ACATGAGCGCGGGCTCCATGAACATGTCGTCGTACGTGGGCGCTGGCAT
GAGCCCGTCCCTGGCGGGGATGTCCCCCGGCGCGGGCGCCATGGCGGGC
ATGGGCGGCTCGGCCGGGGCGGCCGGCGTGGCGGGCATGGGGCCGCAC
TTGAGTCCCAGCCTGAGCCCGCTCGGGGGGCAGGCGGCCGGGGCCATG
GGCGGCCTGGCCCCCTACGCCAACATGAACTCCATGAGCCCCATGTACG
GGCAGGCGGGCCTGAGCCGCGCCCGCGACCCCAAGACCTACAGGCGCA
GCTACACGCACGCAAAGCCGCCCTACTCGTACATCTCGCTCATCACCAT
GGCCATCCAGCAGAGCCCCAACAAGATGCTGACGCTGAGCGAGATCTA
CCAGTGGATCATGGACCTCTTCCCCTTCTACCGGCAGAACCAGCAGCGC
TGGCAGAACTCCATCCGCCACTCGCTCTCCTTCAACGACTGTTTCCTGAA
GGTGCCCCGCTCGCCCGACAAGCCCGGCAAGGGCTCCTTCTGGACCCTG
CACCCTGACTCGGGCAACATGTTCGAGAACGGCTGCTACCTGCGCCGCC
AGAAGCGCTTCAAGTGCGAGAAGCAGCTGGCGCTGAAGGAGGCCGCAG
GCGCCGCCGGCAGCGGCAAGAAGGCGGCCGCCGGAGCCCAGGCCTCAC
AGGCTCAACTCGGGGAGGCCGCCGGGCCGGCCTCCGAGACTCCGGCGG
GCACCGAGTCGCCTCACTCGAGCGCCTCCCCGTGCCAGGAGCACAAGCG
AGGGGGCCTGGGAGAGCTGAAGGGGACGCCGGCTGCGGCGCTGAGCCC
CCCAGAGCCGGCGCCCTCTCCCGGGCAGCAGCAGCAGGCCGCGGCCCA
CCTGCTGGGCCCGCCCCACCACCCGGGCCTGCCGCCTGAGGCCCACCTG
AAGCCGGAACACCACTACGCCTTCAACCACCCGTTCTCCATCAACAACC
TCATGTCCTCGGAGCAGCAGCACCACCACAGCCACCACCACCACCAACC
CCACAAAATGGACCTCAAGGCCTACGAACAGGTGATGCACTACCCCGGC
TACGGTTCCCCCATGCCTGGCAGCTTGGCCATGGGCCCGGTCACGAACA

AAACGGGCCTGGACGCCTCGCCCCTGGCCGCAGATACCTCCTACTACCA
GGGGGTGTACTCCCGGCCCATTATGAACTCCTCTTAA

Supplementary Table S3. Sequencing results of reporter gene vectors

Plasmids	PGL3-basic-H_SIRT1 promoter(-1923to+66) WT rs12778366T

TCATTGATTGGTCAGATGGATTTCAGAGGGATTGGTATGAAGGAACGCTT
CAAAGATTTTTTTTTAATTTAAGTTCCAGGATACAGGTGCAGAATGTGTA
GGTTTGTTACATACTTATAGGTGAGCCATGGTGGTTTGCTGCACCAATCAA
CCCCTCATCTAGGTTTATTTATATTTTTTTTGAGACGGAGTTTCGCTCTT
GCTGCCCAGGCTGGTGTGCAATGGTGCGATCTCGGCTCACCGCAACCTCT
GCCTGCTGGGTTCAAGCGATTCTCCTGCCTCAGCCTTCTGAGTAGCTGGGA
TTACAGGCATGTGCCACCACGCCCGGCTAATTTTGTATTTTTAGTAGAGAC
GGGGTTTCTCCATGTTGGTCAGGCTGGTCTCGAACTTCCGACTTCAGGTGA
TCTGTCCGCCTCGGCCTCCCAAAGTGCTGGGATTACAGGCAGCAGCCACC
GCGCCCAGCCAGGTTTTAAGTCCCGCATGCATTAGCTATTTGTCCTAATGC
TCTCCCTCCCCTTGACCCCCACGCAACCAAAGATGGTTTTAAGGCTTCTAG
GACTGGAGATGATTACTTTCAGAAATGAATAGTGGTGACCAGATGAAATA
AGACTTTTGGATGTAGATAGGACCTTAGCGATATATTTCCAGCTTTTTATT
TTTTATTTTAAGGATGAAGAAACAGCTTTAAGTTGGTTGCCTAAAGTCACG
CAGGTAATTGATGCAGTATGGCCAGAACCCATACTAGGCTTAAGGGACAG
TGTACCATAAACTCGTAGAGGTCCTTTAAACAGATAGAAACGCTGTGCTC
CAGGCAGATGCCATAACAAACACTGGCTCTAGATCTACCATGGGTTATAT
GGGTCCTTAGTTAAGATTAGATATGGAGTCACAGTGTGCCAGAATTTCAG
GGAGAGAGGAAAGTGGAAGGGCTTTCCACTAAGCCTTTTGAACTACTAGG
TACCCCTCGTTTTACATCTGGTTATCTCATTTAAATCTATGACGTTTTAAAA
TACTTATTACCATTTAAGACATGAGAAAAATTAAGTTTAGAAACGGCTAG
ATAGCTCACGCTAGAAAGGAAGGACTCCAAATTTTAACCAAGGGCAGATG
TGCATGGAGGCCAAGTCATTTCCTTCCCATGCTCTCATACTGACCCAACAA
ACCCATTCTGCACGTGAGAAAACTGAGGCCCGGAGGAGGGAATTCACAC
ACGTTTGAAGCCAAGCTGGGGCCAGAAAGTAGATCGGCTGATCTCCAAAC
CTCCACGTCAAAGGTCTTCCCAGGAGGACATATGCCTTCAAGGATTTTAC
AATGTATACCACCCTACAAGTGATGGGAGAGAGGGGAAAAAAGCAACCG
ACTAAGGAGAAAAGCAAGGAGCAGAAAAAGGAGCAAAAGAGGAGCTGT
CAGAACGGTGTGAGGAGAGTGGGAAAGGAGCCGCCTCCTTTTGCCTCTCT
TCCTACTTATTAACAAAACAGAACGACTATCCAACGTATTTCAGGGAGCT
AAGTCTTAGCCAGCTTCAGCTGTGTTTTAACCCTTAGCTAAATATAGACAA
GGCTAAGGCAGGCCAGGTGTACACTTCAGGAAGACGTGGAAATTCCCAG
GGCGGACCAAAACTTGAGCTGTTCCGGCGGTAGTGATTTGAGGTCAGTTT
GAAAGAGAAGTTGAGAAAGCGGCCGAGGGGCGAATTTGGCTGCACTACA
CGCTCGCCACAAAGAGGAAGGGCCGCCGGCCGCCGGGGCCGAGTGCGCT
TCCAGCCCAGGCGGAGCGGTAGACGCAACAGCCTCCGCCCGCCACGTGAC
CCGTAGTGTTGTGGTCTGGCCCGCGTGGGTGGCGGGAGCGCCGAGAGGGC
GGGGGCGGCGATGGGGCGGGTCACGTGATGGGGTTTAAATCTCCCGCAGC

CGGAGCCGCGGGGGCGCCAGTGCCGCGCGTCGAGCGGGAGCAGAGGAGG
CGAGGGAGGAGGGCCAGAGAGGCAGTTGGAAG

special circumstances	none
Plasmids	PGL3-basic-H_SIRT1 promoter(-1923to+66) MT rs12778366C

TCATTGATTGGTCAGATGGATTTCAGAGGGATTGGTATGAAGGAACGCTT
CAAAGATTTTTTTTTTAATTTAAGTTCCAGGATACAGGTGCAGAATGTGTA
GGTTTGTTACATACTTATAGGTGAGCCATGGTGGTTTGCTGCACCAATCAA
CCCCTCATCTAGGTTTTATTTATATTTTTTTTTGAGACGGAGTTTCGCTCTT
GCTGCCCAGGCTGGTGTGCAATGGTGCGATCTCGGCTCACCGCAACCTCT
GCCTGCTGGGTTCAAGCGATTCTCCTGCCTCAGCCTTCTGAGTAGCTGGGA
TTACAGGCATGTGCCACCACGCCCGGCTAATTTTGTATTTTTAGTAGAGAC
GGGGTTTCTCCATGTTGGTCAGGCTGGTCTCGAACTTCCGACTTCAGGTGA
TCTGTCCGCCTCGGCCTCCCAAAGTGCTGGGATTACAGGCAGCAGCCACC
GCGCCCAGCCAGGTTTTAAGTCCCGCATGCATTAGCTATTTGTCCTAATGC
TCTCCCTCCCCTTGACCCCCACGCAACCAAAGATGGTTTTAAGGCTTCTAG
GACTGGAGATGATTACTTTCAGAAATGAACAGTGGTGACCAGATGAAATA
AGACTTTTGGATGTAGATAGGACCTTAGCGATATATTTCCAGCTTTTTATT
TTTTATTTTAAGGATGAAGAAACAGCTTTAAGTTGGTTGCCTAAAGTCACG
CAGGTAATTGATGCAGTATGGCCAGAACCCATACTAGGCTTAAGGGACAG
TGTACCATAAACTCGTAGAGGTCCTTTAAACAGATAGAAACGCTGTGCTC
CAGGCAGATGCCATAACAAACACTGGCTCTAGATCTACCATGGGTTATAT
GGGTCCTTAGTTAAGATTAGATATGGAGTCACAGTGTGCCAGAATTTCAG
GGAGAGAGGAAAGTGGAAGGGCTTTCCACTAAGCCTTTTGAACTACTAGG
TACCCCTCGTTTTACATCTGGTTATCTCATTTAAATCTATGACGTTTTAAAA
TACTTATTACCATTTAAGACATGAGAAAAATTAAGTTTAGAAACGGCTAG
ATAGCTCACGCTAGAAAGGAAGGACTCCAAATTTTAACCAAGGGCAGATG
TGCATGGAGGCCAAGTCATTTCCTTCCCATGCTCTCATACTGACCCAACAA
ACCCATTCTGCACGTGAGAAAACTGAGGCCCGGAGGAGGGAATTCACAC
ACGTTTGAAGCCAAGCTGGGGCCAGAAAGTAGATCGGCTGATCTCCAAAC
CTCCACGTCAAAGGTCTTCCCAGGAGGACATATGCCTTCAAGGATTTTAC
AATGTATACCACCCTACAAGTGATGGGAGAGAGGGGAAAAAGCAACCG
ACTAAGGAGAAAAGCAAGGAGCAGAAAAAGGAGCAAAAGAGGAGCTGT
CAGAACGGTGTGAGGAGAGTGGGAAAGGAGCCGCCTCCTTTTGCCTCTCT
TCCTACTTATTAACAAAACAGAACGACTATCCAACGTATTTCAGGGAGCT
AAGTCTTAGCCAGCTTCAGCTGTGTTTTAACCCTTAGCTAAATATAGACAA
GGCTAAGGCAGGCCAGGTGTACATCATTGATTGGTCAGATGGATTTCAGA
GGGATTGGTATGAAGGAACGCTTCAAAGATTTTTTTTTTAATTTAAGTTCC
AGGATACAGGTGCAGAATGTGTAGGTTTGTTACATACTTATAGGTGAGCC
ATGGTGGTTTGCTGCACCAATCAACCCCTCATCTAGGTTTTATTTATATTTT
TTTTTGAGACGGAGTTTCGCTCTTGCTGCCCAGGCTGGTGTGCAATGGTGC
GATCTCGGCTCACCGCAACCTCTGCCTGCTGGGTTCAAGCGATTCTCCTGC

	CTCAGCCTTCTGAGTAGCTGGGATTACAGGCATGTGCCACCACGCCCGGC<br>TAATTTTGTATTTTTAGTAGAGACGGGGTTTCTCCATGTTGGTCAGGCTGG<br>TCTCGAACTTCCGACTTCAGGTGATCTGTCCGCCTCGGCCTCCCAAAGTGC<br>TGGGATTACAGGCAGCAGCCACCGCGCCCAGCCAGGTTTTAAGTCCCGCA<br>TGCATTAGCTATTTGTCCTAATGCTCTCCCTCCCCTTGACCCCCACGCAAC<br>CAAAGATGGTTTTAAGGCTTCTAGGACTGGAGATGATTACTTTCAGAAAT<br>GAACAGTGGTGACCAGATGAAATAAGACTTTTGGATGTAGATAGGACCTT<br>AGCGATATATTTCCAGCTTTTTATTTTTTATTTTAAGGATGAAGAAACAGC<br>TTTAAGTTGGTTGCCTAAAGTCACGCAGGTAATTGATGCAGTATGGCCAG<br>AACCCATACTAGGCTTAAGGGACAGTGTACCATAAACTCGTAGAGGTCCT<br>TTAAACAGATAGAAACGCTGTGCTCCAGGCAGATGCCATAACAAACACTG<br>GCTCTAGATCTACCATGGGTTATATGGGTCCTTAGTTAAGATTAGATATGG<br>AGTCACAGTGTGCCAGAATTTCAGGGAGAGAGGAAAGTGGAAGGGCTTT<br>CCACTAAGCCTTTTGAACTACTAGGTACCCCTCGTTTTACATCTGGTTATC<br>TCATTTAAATCTATGACGTTTTAAAATACTTATTACCATTTAAGACATGAG<br>AAAAATTAAGTTTAGAAACGGCTAGATAGCTCACGCTAGAAAGGAAGGA<br>CTCCAAATTTTAACCAAGGGCAGATGTGCATGGAGGCCAAGTCATTTCCT<br>TCCCATGCTCTCATACTGACCCAACAAACCCATTCTGCACGTGAGAAAAC<br>TGAGGCCCGGAGGAGGGAATTCACACACGTTTGAAGCCAAGCTGGGGCC<br>AGAAAGTAGATCGGCTGATCTCCAAACCTCCACGTCAAAGGTCTTCCCAG<br>GAGGACATATGCCTTCAAGGATTTTACAATGTATACCACCCTACAAGTGA<br>TGGGAGAGAGGGGAAAAAAGCAACCGACTAAGGAGAAAAGCAAGGAGC<br>AGAAAAAGGAGCAAAAGAGGAGCTGTCAGAACGGTGTGAGGAGAGTGG<br>GAAAGGAGCCGCCTCCTTTTGCCTCTCTTCCTACTTATTAACAAAACAGAA<br>CGACTATCCAACGTATTTCAGGGAGCTAAGTCTTAGCCAGCTTCAGCTGTG<br>TTTTAACCCTTAGCTAAATATAGACAAGGCTAAGGCAGGCCAGGTGTACA<br>CTTCAGGAAGACGTGGAAATTCCCAGGGCGGACCAAAACTTGAGCTGTTC<br>CGGCGGTAGTGATTTGAGGTCAGTTTGAAAGAGAAGTTGAGAAAGCGGCC<br>GAGGGGCGAATTTGGCTGCACTACACGCTCGCCACAAAGAGGAAGGGCC<br>GCCGGCCGCCGGGGCCGAGTGCGCTTCCAGCCCAGGCGGAGCGGTAGAC<br>GCAACAGCCTCCGCCCGCCACGTGACCCGTAGTGTTGTGGTCTGGCCCGC<br>GTGGGTGGCGGGAGCGCCGAGAGGGCGGGGGCGGCGATGGGGCGGGTCA<br>CGTGATGGGGTTTAAATCTCCCGCAGCCGGAGCCGCGGGGGCGCCAGTGC<br>CGCGCGTCGAGCGGGAGCAGAGGAGGCGAGGGAGGAGGGCCAGAGAGG<br>CAGTTGGAAG
special circumstances	The mutation sequences are shown in bold and underlined.

Supplementary Table S4. Cell transfection system

reagents	volumetric
Serum-free DMEM	50µl

reagents	volumetric
Gene Overexpression Plasmids（OE/NC）	450ng
promoter reporter gene plasmid（Pro/NC）	75 ng
pRL-TK	25ng
HG transgene reagent	1.5μg

Supplementary Table S5. Basic Characteristics of the Study Population

variant	experimental group (n = 639)		control group (n =675)		*P*
	n	%	n	%	
Age (mean ± standard deviation)[a]	39.84±7.89		39.99±7.89		0.740
gender[b]					0.706
male	594	93.0	631	93.5	
female	45	7.0	44	6.5	
smoking[b]					0.322
all the time	355	55.6	348	51.6	
Occasionally	31	4.9	39	5.8	
never	253	39.6	288	42.7	
drinking[b]					0.933
all the time	196	30.7	212	31.4	
Occasionally	85	13.3	86	12.7	
never	358	56.0	377	55.9	
Noise duration (mean ± standard deviation)[a]	18.41±7.65		18.06±7.75		0.486

variant	experimental group (n = 639)		control group (n =675)		*P*
	n	%	n	%	
Decibels (mean ± standard deviation) [b]	87.05±7.75		87.67±7.64		0.214
High frequency hearing threshold (mean ± standard deviation)[b]	37.78±12.11		19.50±8.65		**<0.001**

[a] two-sided t-test;

[b] two-sided χ2 test

Supplementary Table S6. Basic information about SNPs sites and Hardy-Weinberg tests

gene	loci	chromosomes	Functional sequence	MAF			P for HWE[b]
				Control	Case	databases[a]	
SIRT1	rs10997868	10:67905202	intro variant	0.167	0.173	0.50	0.97
	rs12049646	10:67864226	intergenic variant	0.277	0.283	0.36	0.72
	rs12778366	10:67883321	intergenic variant	0.095	0.127	0.19	0.24
	rs2236319	10:67889462	intron variant	0.284	0.277	0.37	0.77
	rs2273773	10:67906841	synonymous variant	0.284	0.277	0.37	0.75
	rs3758391	10:67883584	intergenic variant	0.173	0.167	0.47	0.88
	rs7895833	10:67863299	intergenic variant	0.266	0.296	0.49	0.38

[a] Data from NCBI dbSNP database

[b] *P*-value for Hardy-Weinberg test

Supplementary Table S7. Genotype distributions of the seven candidate SNPs in NIHL cases and controls

gene	loci	alleles	case group	control group	Unadjusted *P* value[a]	Adjusted *P* value[b]
		**A1/A2**	**A1A1/A1A2/A2A2**			
SIRT1	rs10997868	A/C	15/187/437	22/188/465	0.546	>0.999
	rs12049646	C/T	41/257/341	52/278/345	0.559	>0.999
	rs12778366	C/T	8/146/485	2/124/549	**0.014**	**0.041**
	rs2236319	G/A	42/255/342	52/279/344	0.559	>0.999
	rs2273773	C/T	42/255/342	52/280/343	0.536	>0.999
	rs3758391	C/T	15/187/437	22/189/464	0.560	>0.999
	rs7895833	A/G	45/286/308	48/263/364	0.093	>0.999

[a] Genotype distributions were compared using Pearson's chi-square test, except for rs12778366, for which the Fisher-Freeman-Halton exact test was used because one expected cell count was below 5.

[b]To account for the strong correlation among the seven SIRT1 SNPs, the effective number of independent tests was estimated using the eigenvalue-based method. The effective number of tests was 3.

Supplementary Table S8. Association between rs12778366 and NIHL susceptibility under different genetic models

Genetic model	Genotypes	Case group n(%)	Control group n(%)	P^a	P^b	Adjusted OR (95% CI)[b]
Codominant	TT	485(75.90)	549 (81.33)	**0.015**		
	CT	146(22.85)	124(18.37)		**0.039**	1.328 (1.014-1.740)
	CC	8(1.25)	2(0.30)		0.056	4.548 (0.959-21.568)
Dominant	TT	485(75.90)	549(81.33)	**0.016**		1.00(Ref)
	CT / CC	154(24.10)	126(18.67)		**0.018**	1.380 (1.057-1.801)
Recessive	TT / CT	631(98.75)	673 (99.70)	**0.046**		1.00(Ref)
	CC	8(1.25)	2(0.30)		0.066	4.298 (0.907-20.364)
Alleles	T	1116 (87.32)	1222(90.52)	**0.004**		1.00(Ref)
	C	162(12.68)	128(9.48)		**0.009**	1.386 (1.084-1.772)

[a] By two-sided χ2 test；

[b] Adjusted for age, sex, smoking and alcohol consumption

Supplementary Table S9. Stratified analyses of the association between gene polymorphisms and susceptibility to NIHL (dominant model)

**Rs12778366**						
**hierarchical variable**	**TT(NIHL/ control)**		**CT/CC(NIHL/ control)**		P^a	**OR (CI95%)**[a]
	**quantities**	**component ratio**	**quantities**	**component ratio**		
Noise work experience (years)						
≤15	140/170	35.9/43.6	37/43	9.5/11.0	0.862	1.045 (0.638-1.711)
>15	210/256	36.0/43.8	75/43	12.8/7.4	**<0.001**	2.126 (1.401-3.226)

Rs12778366						
hierarchical variable	TT(NIHL/ control)		CT/CC(NIHL/ control)		P^a	OR (CI95%)[a]
	quantities	component ratio	quantities	component ratio		
noise intensity [dB(A)]						
≤85.0	158/175	38.9/43.1	42/31	10.3/7.6	0.118	1.501 (0.900-2.503)
>85.0	192/251	33.8/44.2	70/55	12.3/9.7	**0.012**	1.664 (1.115-2.482)

[a] By two-sided $\chi 2$ test

Supplementary Table S10. Interactions between gene polymorphisms and environmental factors

variability	Genotype	NIHL	%	control group	%	P^a	OR (CI95%)[a]
Noise work experience (years)							
≤15	TT	140	30.5	170	31.9		1.00(Ref)
≤15	CC+TC	37	7.9	43	10.2	0.942	1.019（0.620-1.674）
>15	TT	210	45.9	256	48.1	0.548	1.103（0.801-1.520）
>15	CC+TC	75	15.7	43	9.8	**<0.001**	2.353（1.484-3.730）
noise intensity[dB(A)]							
≤85	TT	158	34.7	175	32.9		1.00(Ref)
≤85	CC+TC	42	8.1	31	7.3	0.115	1.511（0.904-2.525）
>85	TT	192	42.1	251	47.2	0.148	0.805（0.600-1.080）
>85	CC+TC	70	15.1	55	12.6	0.182	1.332（0.875-2.030）

[a] Adjusted for age, sex, smoking and alcohol consumption. TT is the reference genotype for all OR calculations.

Supplementary Table S11. SNPs interaction analysis

model	Training Bal Acc	Testing Bal Acc	Sign Test (*P*)	CV Consistency
Expose level with noise	0.5879	0.5596	10 (**0.0010**)	10/10
Work time with noise Expose level with noise	0.7009	0.5086	5 (0.6230)	10/10
Work time with noise Expose level with noise rs 12049646	0.7649	0.5092	5 (0.6230)	9/10
Drinking Work time with noise Expose level with noise rs 12049646	0.8149	0.5071	4(0.8281)	8/10
Smoking Drinking Work time with noise Expose level with noise rs 2236319	0.8476	0.5265	5(0.6230)	5/10
